# Identifying the Main Bottlenecks in the Workflow of Saudi Arabian Emergency Departments (EDs)

**DOI:** 10.1155/jonm/4239274

**Published:** 2025-02-24

**Authors:** Abdulellah Al Thobaity

**Affiliations:** Department of Medical Surgical Nursing, College of Nursing, Taif University, Taif, Saudi Arabia

**Keywords:** bottleneck analysis and Saudi Arabia healthcare, emergency department overcrowding, healthcare efficiency, workflow optimization

## Abstract

**Background:** Crowded emergency departments (EDs) adversely affect patient care and healthcare efficiency, leading to prolonged wait times, delayed treatments, and increased medical errors. This issue also diminishes patient satisfaction and disrupts hospital operations. In Saudi Arabia, ED overcrowding impacts response times and staff morale, highlighting the need for efficient patient flow processes to ensure timely and effective care.

**Objectives:** The aim of this study is to identify the main bottlenecks in the workflow of Saudi Arabian EDs from patient arrival to disposition.

**Design:** A retrospective quantitative study analyzed data from 753 patients across multiple hospitals in Saudi Arabia's EDs to identify workflow bottlenecks. Using SPSS and AMOS for data analysis, various statistical methods, including ANOVA and structural equation modeling (SEM), were employed to evaluate key performance metrics and their influence on the total length of stay (LOS).

**Results:** The Doctor to Decision Time is the most significant bottleneck, followed by the Triage to Doctor Time. CTAS3 and CTAS4 categories experience the most significant delays across multiple stages. In addition, the default model in AMOS 29 shows an excellent fit, indicating that reducing delays in Decision to Disposition Time (estimate = 0.840) and Doctor to Decision Time (estimate = 0.442) is crucial for improving the total LOS in the ED.

**Conclusion:** This study identifies significant inefficiencies in the ED workflow in Saudi Arabia, particularly in the Doctor to Decision Time and Triage to Doctor Time stages, and recommends streamlining consultation protocols, enhancing medication delivery, expediting lab and radiology services, and increasing staffing to improve operational efficiency and patient outcomes. Faster bed turnover reduces Decision to Disposition Time and frees up ED beds. Adequate staffing improves triage, evaluation times, and care quality. Well-trained nurses enhance patient interactions and reduce delays. Standardized guidelines ensure timely treatment. Effective communication and teamwork improve patient flow and reduce bottlenecks.

## 1. Introduction

The crowded emergency departments (EDs) pose significant issues due to their adverse effects on patient care and overall healthcare system efficiency. Overcrowding leads to prolonged wait times, which can delay critical treatments, thereby increasing patient suffering and the risk of complications [1, 2]. In addition, the strain on resources and personnel in a crowded ED heightens the likelihood of medical errors, compromising patient safety and outcomes [3, 4]. This environment also diminishes patient satisfaction, as individuals experience longer lengths of stay (LOS) and a perceived decline in the quality of care. Moreover, overcrowded EDs can disrupt the flow of the entire hospital, affecting not only those in the ED but also patients awaiting elective procedures or transfers from other departments [1, 3–5]. Consequently, ED crowding is a multifaceted problem that underscores the need for systemic solutions to improve patient care, streamline operations, and enhance overall healthcare delivery. In Saudi Arabia, the ED is crucial to the success of healthcare facilities, but overcrowding significantly impacts the quality of care and leads to patient dissatisfaction. This overcrowding not only affects the hospital's response time due to long queues but also negatively influences employee morale and job satisfaction, as healthcare professionals face increased workloads and frustration. In any ED, the patient goes through a process known as patient flow. The process of patient flow in EDs in Saudi Arabia involves several critical stages designed to ensure timely and effective care.

**Figure 1:**
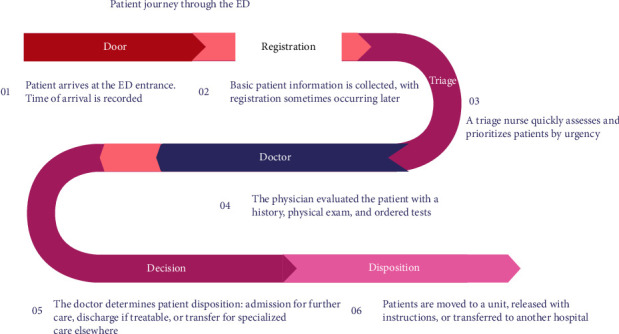
Patient journey through the emergency department (ED).

The patient flow in EDs in Saudi Arabia is illustrated in Figure 1, highlighting the key stages and decision points involved in providing efficient and effective care. The patient's journey begins at the ED entrance, where their arrival time is recorded, marking the official start of their ED experience. This initial timestamp is crucial for tracking patient wait times and overall ED efficiency. Basic patient information is then collected, including name, date of birth, insurance details, and contact information. This registration process may occur at the entrance or later in the flow, depending on the ED's workflow. The goal is to gather essential data for patient identification, billing, and communication [1, 4].

A triage nurse then conducts a quick assessment of the patient's condition, evaluating their chief complaint, vital signs, and overall appearance. Based on this assessment, the nurse assigns a triage level, which prioritizes patients based on the urgency of their condition. This system ensures that patients with the most critical needs receive immediate attention. The physician then evaluates the patient, taking a detailed medical history, conducting a physical exam, and ordering necessary tests. This comprehensive assessment helps determine the nature and severity of the patient's condition. Based on the physician's evaluation, a decision is made regarding the patient's disposition. This involves choosing one of three options: admission, discharge, or transfer. If the patient requires further care, they are admitted to the hospital for ongoing treatment. If their condition is treatable in the ED, they are discharged with instructions for follow-up care. If the patient needs specialized care that is not available at the ED, they are transferred to another facility equipped to handle their specific needs [4, 6]. Finally, the patient is moved according to the doctor's decision. This involves transferring them to a specific unit within the hospital, releasing them from the ED with instructions for follow-up care, or transporting them to another hospital for specialized care. Overall, Figure 1 highlights the importance of a structured and efficient process for managing patients in an emergency setting. It emphasizes the crucial roles of triage, physician evaluation, and decision-making in ensuring that patients receive timely and appropriate care.

From an international perspective, the LOS in EDs serves as a critical metric of operational efficiency and quality of patient care, influenced by numerous factors extensively analyzed across various studies. Detailed examinations of ED processes reveal that specific care intervals, such as diagnostic testing and specialist consultations, significantly extend LOS, particularly for patients in intermediate triage levels, emphasizing the need for targeted interventions to streamline these processes and reduce delays [7]. The profound impact of LOS on patient outcomes, particularly in-hospital mortality (IHM), has been further elucidated, showing a statistically significant correlation between prolonged LOS (exceeding 24 h) and increased IHM for ICU-admitted patients, while shorter EDLOS is associated with lower mortality in non-ICU patients, indicating that both extremes of LOS can adversely affect patient health outcomes [8]. Moreover, an exploration of hospital-at-home interventions as alternatives to traditional in-hospital care for chronic disease patients demonstrates that such interventions can reduce LOS and lower the risk of long-term care admissions and readmissions, presenting a viable substitute that alleviates the burden on EDs and enhances patient satisfaction and outcomes [9]. In addition, global strategies to measure and improve ED performance have been reviewed, identifying a diverse array of interventions aimed at reducing LOS, including changes to triage processes, care transitions, and team composition, highlighting the multifaceted nature of ED performance improvement efforts and the necessity for a holistic approach encompassing time efficiency, process efficacy, cost, and clinical outcomes [10]. Collectively, these studies illuminate the global challenges and strategies associated with managing LOS in EDs, emphasizing the importance of efficient process management, the adoption of alternative care models, and comprehensive performance measurement systems to optimize patient care and operational efficiency in emergency settings.

In Saudi Arabia, the LOS in EDs is significantly influenced by factors such as shift time, season, severity level, and hospital type, with notable improvements observed over the study period [6]. Older aged females, those discharged against medical advice, and patients requiring emergent and urgent care are the primary predictors of prolonged stay in the ED of Saudi Arabia [11]. Morning arrival and presentation during summertime are key predictors of prolonged LOS in the pediatric ED, with systemic factors like staffing and infrastructure being modifiable elements that could influence these outcomes [12]. The increased LOS for patients in the ED is associated with the crowding status of the ED, necessitating measures and interventions by the Ministry of Health to address and reduce crowding [13]. Nonurgent and returned visits are due to a lack of knowledge about primary health care (PHC), triage, and telemedicine services, while prolonged LOS is caused by slow bed turnover, laboratory and consultation delays, and slow response to final decisions, leading to staff burnout, wrong diagnoses, and poor management plans [5]. The study identified critical areas of waste affecting patient flow in the ED, including issues related to quality management, facilities, patients, staff, administration, data, and work schedule changes, and recommended designing a lean process flow to eliminate these root causes and improve efficiency [14]. Encourage policymakers in each ED in Saudi Arabia to improve waiting time and LOS through continuous evaluation, standardized systems, AI-enhanced triaging, and senior physician involvement, while also ensuring 24/7 PHC centers for Canadian Triage and Acuity Scale (CTAS) Level 4 and 5 referrals [4].

ED bottlenecks are a global healthcare challenge, with various countries implementing innovative solutions. In Canada, researchers combined benchmarking and the design of experiments within simulation modeling to identify key issues in patient flow, particularly in treatment procedures and emergency room holding [15]. The United States focused on improving patient throughput by introducing a flow nurse coordinator role, which successfully reduced transfer times by 20% [16]. In the United Kingdom, a study of a Level 1 ED revealed opportunities to improve patient throughput by reducing repeat tests and better incorporating operational complexity into ED processes [17]. Meanwhile, in Saudi Arabia, researchers analyzed patient flow for those with abdominal pain, identifying major bottlenecks in diagnostic delays, multispecialist decision-making, and departure processes [18]. These diverse approaches from different countries highlight the complexity of ED bottlenecks and the need for tailored solutions to enhance healthcare delivery worldwide.

Despite extensive research on the LOS in EDs and the factors influencing it, there remains a critical gap in addressing the specific stages of the patient journey that contribute most significantly to delays within the context of Saudi Arabia. The current literature often lacks a detailed examination of performance metrics across different triage categories in Saudi EDs and how these metrics impact overall patient flow and outcomes. This gap underscores the need for targeted interventions at identified bottlenecks within the ED process in Saudi hospitals, particularly at stages where delays are most pronounced. Addressing these specific inefficiencies is crucial for optimizing both operational efficiency and patient care quality in emergency settings in Saudi Arabia. Therefore, the aim of this study is to identify the main bottlenecks in the workflow of Saudi Arabian EDs from patient arrival to disposition. Specifically, the study seeks to determine the stages in the ED workflow where delays are most pronounced and to analyze how these delays vary across different patient triage categories.

## 2. Materials and Methods

### 2.1. Design

A quantitative design using retrospective data analysis was employed to identify and evaluate bottlenecks in the ED in Saudi Arabia.

### 2.2. Setting and Population

A sample of 753 patients who visited the ED within a 3-month period was collected from each hospital database. Specifically, data from 340 patients (25% of the total sample) were collected for each hospital for the period between December 2019 and February 2020. These patients were categorized from Level 1 to Level 5 based on the CTAS.

### 2.3. Data Collection Methods

Ethical approval was secured from the Directorate of Health Affairs in Jeddah. Patient consent was deemed unnecessary as the researcher utilized retrospective data solely concerning the timing of patient arrivals, without accessing any demographic or personal information. All data were maintained and stored in alignment with Taif University's protocol on patient confidentiality, ensuring compliance with the Declaration of Helsinki. Data were exported from the database of each hospital, previously collected by the key performance indicator (KPI) collectors in the ED of each hospital. These KPI collectors gather data yearly and save it in special databases and/or Excel files. The data were gathered manually from patient records and/or electronically from the hospital databases.

### 2.4. Bottleneck Calculation Methods

The Saudi Ministry of Health employs Ada'a to monitor KPIs, enabling hospitals to assess and enhance their performance. Among these KPIs are three critical time measurements in emergency care: Door (registration or triage) to Doctor Time, Doctor to Decision Time, and Decision to Disposition Time. These metrics collectively provide insight into the efficiency of patient flow through EDs, from initial entry to final disposition [19].  Table 1 outlines the methods used to measure bottlenecks in Saudi Arabian EDs by tracking four key metrics: Registration to Triage Time, Triage to Doctor Time, Doctor to Decision Time, and Decision to Disposition Time. Each metric has defined starting and ending points, with the duration calculated in minutes by subtracting the starting point from the ending point. Performance is categorized into four levels: World Class, Acceptable, Needs Improvement, and Unacceptable, based on specific time frames. Notably, in some cases, triage and physician contact may precede patient registration, which is accounted for in the measurements. These metrics help identify areas needing improvement to enhance efficiency and patient care in EDs.

### 2.5. Statistical Analysis

Data analysis was conducted using SPSS Version 29 and AMOS 29. Descriptive statistics were employed to summarize performance metrics at each stage of the patient journey through the ED. One-way ANOVA was used to compare times between different triage categories (CTAS1 to CTAS5) across various stages, identifying significant differences in Registration to Triage, Triage to Doctor, Doctor to Decision, and Decision to Disposition Times. Structural equation modeling (SEM) was performed in AMOS 29 to assess the impact of four predictor variables (Registration to Triage Time, Triage to Doctor Time, Doctor to Decision Time, and Decision to Disposition Time) on the outcome variable, total LOS. Model fit was evaluated using NFI, RFI, IFI, TLI, and CFI. Standardized regression weights were calculated to determine the strength and direction of relationships between predictor and outcome variables, providing insights into each stage's impact on total LOS.

## 3. Results

Based on the analysis of performance metrics at each stage of the patient journey through the ED, we have identified several critical bottlenecks. The data, summarized in Table 2, highlight the stages that require urgent attention and improvement. The Doctor to Decision Time stage represents the most significant bottleneck in the patient journey. According to Table 2, a staggering 86.1% of the times are rated as “Unacceptable.” This indicates severe delays in the process where doctors make decisions regarding patient care. Efforts should be concentrated on streamlining decision-making processes to reduce these delays and improve overall patient outcomes. The Triage to Doctor Time is the second major bottleneck, with 48.5% of the times in this stage rated as “Unacceptable.” This highlights a substantial inefficiency in moving patients from triage to a doctor's attention. Enhancing the efficiency of this transition is crucial to reduce waiting times and improve patient flow. Although not as severe as the subsequent stages, the Registration to Triage process also shows room for improvement, with 36.0% of the cases falling under “Late Registration.” Addressing these delays can ensure that patients are triaged promptly, setting a better pace for the subsequent stages of care. The Decision to Disposition Time stage shows relatively better performance, with nearly half of the times rated as “World Class” (48.2%), but it still has significant delays, with 34.1% of the times rated as “Unacceptable.” Reducing these delays can further enhance the efficiency of patient throughput toward discharge or further treatment.

The analysis of the patient journey times in the ED, categorized by triage levels (CTAS1 to CTAS5), highlights several critical bottlenecks, as summarized in Table 3. The most significant delays are observed in CTAS3 and CTAS4 across all stages. In the Registration to Triage stage, 15.9% of CTAS3 cases and 9.2% of CTAS4 cases registered late. In the Triage to Doctor Time stage, 21.1% of CTAS3 and 20.8% of CTAS4 cases were rated as “Unacceptable.” The Doctor to Decision Time stage shows the most severe delays, with 42.0% of CTAS3 and 26.8% of CTAS4 cases being “Unacceptable.” Finally, in the Decision to Disposition Time stage, 15.8% of CTAS3 and 7.0% of CTAS4 cases fall under the “Unacceptable category.” The data indicate that CTAS3 consistently has the highest total cases across all stages, emphasizing significant inefficiencies. These findings suggest that targeted improvements in these categories, particularly in the Doctor to Decision Time stage, are essential to enhance the overall efficiency and patient flow in the ED.

The ANOVA indicates that there is a statistically significant difference in Registration to Triage Times among the different triage categories (*F* (4, 748) = 5.017, *p* < 0.001). As shown in Table 4, the mean difference in Registration to Triage Times between CTAS1 and CTAS2 is −24.00152 min, with a standard error of 6.58768. This difference is statistically significant (*p*=0.003), and the 95% confidence interval ranges from −42.0151 to −5.9879 min. The mean difference between CTAS1 and CTAS3 is −21.22910 min, with a standard error of 5.31061. This difference is highly significant (*p* < 0.001), with a 95% confidence interval of −35.7507 to −6.7075 min. The mean difference between CTAS1 and CTAS4 is −22.40995 min, with a standard error of 5.44842. This difference is also highly significant (*p* < 0.001), with a 95% confidence interval of −37.3083 to −7.5116 min. These findings indicate that CTAS2, CTAS3, and CTAS4 have significant delays compared to CTAS1, suggesting bottlenecks in the Registration to Triage process for these categories, while CTAS5 does not exhibit significant variation in comparison.

The ANOVA in Table 5 highlights significant differences in Triage to Doctor Times among various triage categories. Specifically, CTAS4 experiences notably longer delays compared to CTAS1, with a mean difference of −56.62140 min (*p* < 0.001), with a 95% confidence interval of −88.3553 to −24.8875 min. Similarly, CTAS2 shows significant delays when compared to both CTAS3 and CTAS4, with mean differences of −49.07644 min (*p* < 0.001, 95% CI: −76.0996 to −22.0533 min) and −76.52595 min (*p* < 0.001, 95% CI: −104.4642 to −48.5877 min), respectively. In addition, CTAS2 vs. CTAS5 reveals a significant mean difference of −50.82500 min (*p* < 0.001, 95% CI: −85.5639 to −16.0861 min). The comparison between CTAS3 and CTAS4 also indicates substantial delays, with a mean difference of −27.44951 min (*p* < 0.001, 95% CI: −43.7307 to −11.1684 min). These findings suggest that CTAS4 and CTAS5 are critical bottlenecks in the Triage to Doctor process, highlighting the need for targeted interventions to reduce waiting times and improve patient flow in these categories.

The ANOVA in Table 6 reveals significant differences in Doctor to Decision Times across various triage categories. Specifically, CTAS1 vs. CTAS5 shows a mean difference of 76.22017 min (*p*=0.005), indicating that CTAS5 patients experience substantially longer times. Similarly, CTAS3 vs. CTAS5 has a significant mean difference of 52.32597 min (*p*=0.005). The comparison between CTAS3 and CTAS4 shows a mean difference of 25.94220 min (*p*=0.047). Conversely, comparisons such as CTAS4 vs. CTAS3 and CTAS5 vs. CTAS1 show negative mean differences of −25.94220 min (*p*=0.047) and −76.22017 min (*p*=0.005), respectively, indicating shorter times for the latter categories. In addition, CTAS5 vs. CTAS3 reveals a mean difference of −52.32597 min (*p*=0.005). These findings suggest that CTAS5, in particular, faces significant delays in Doctor to Decision Times, highlighting the need for process improvements to expedite decision-making for these patients.

The ANOVA presented in Table 7 indicates a statistically significant difference in Decision to Disposition Times among the different triage categories (*F* (4, 748) = 25.243, *p* < 0.001). The mean differences highlight that CTAS1 and CTAS2 have significantly shorter Decision to Disposition Times compared to CTAS3, CTAS4, and CTAS5. For example, the mean difference between CTAS1 and CTAS3 is 161.25026 min (*p* < 0.001, 95% CI: 73.3867 to 249.1138 min), between CTAS1 and CTAS4 is 206.05111 min (*p* < 0.001, 95% CI: 115.9077 to 296.1946 min), and between CTAS1 and CTAS5 is 211.16477 min (*p* < 0.001, 95% CI: 103.6230 to 318.7065 min). Similarly, the mean difference between CTAS2 and CTAS3 is 196.95632 min (*p* < 0.001, 95% CI: 120.1943 to 273.7183 min), between CTAS2 and CTAS4 is 241.75717 min (*p* < 0.001, 95% CI: 162.3956 to 321.1187 min), and between CTAS2 and CTAS5 is 246.87083 min (*p* < 0.001, 95% CI: 148.1914 to 345.5503 min). These findings, as detailed in Table 7, suggest that CTAS3, CTAS4, and CTAS5 are primary bottlenecks in the Decision to Disposition process, indicating substantial delays that need to be addressed to improve patient flow and reduce overall waiting times in the ED.

**Figure 2:**
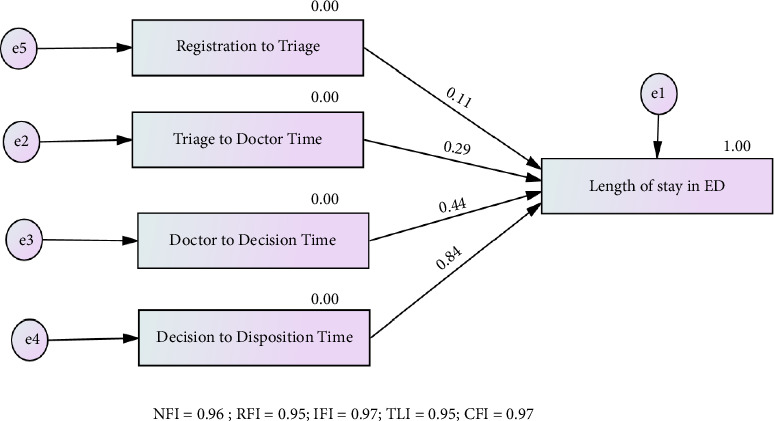
A path analysis of emergency department length of stay.

The fit indices for the default model, generated through AMOS 29 (as shown in Figure 2), are all very close to 1, indicating an excellent fit relative to the independence model. High values for NFI, RFI, IFI, TLI, and CFI suggest the default model fits the data well and explains a substantial portion of the variance. Standardized regression weights (Figure 2) demonstrate the impact of various stages on the total LOS in the ED. Decision to Disposition Time has the strongest positive relationship with LOS (estimate = 0.840), highlighting it as the primary bottleneck. Doctor to Decision Time also significantly affects LOS (estimate = 0.442), representing a secondary bottleneck. Triage to Doctor Time and Registration to Triage Time have weaker positive relationships (estimates = 0.287 and 0.109, respectively). The findings indicate that reducing delays in Decision to Disposition Time should be prioritized for significant improvements. Addressing delays in Doctor to Decision Time is also important. Optimizing these critical bottlenecks will likely yield the most significant reductions in overall LOS in the ED.

## 4. Discussion

The findings of this study illuminate critical inefficiencies within the ED workflow in Saudi Arabia, with significant delays identified in the Doctor to Decision Time and Triage to Doctor Time stages. The Doctor to Decision Time stage is highlighted as the primary bottleneck, with 86.1% of cases rated as “Unacceptable” and a standardized regression weight of 0.840. Similarly, Triage to Doctor Time shows substantial delays, with 48.5% of cases rated as “Unacceptable” and a standardized regression weight of 0.287. Analysis using ANOVA revealed statistically significant differences across triage categories, with CTAS5 patients experiencing the longest delays, indicating that higher severity cases (CTAS3, CTAS4, and CTAS5) contribute significantly to prolonged ED stays. Regression analysis further underscored the impact of Decision to Disposition Time and Doctor to Decision Time on total LOS, with standardized regression weights of 0.442 and 0.840, respectively. These results suggest that prolonged physician decision times, consulting services, medication administration, laboratory and radiological investigations, and resource constraints are major factors driving these delays [20]. To address these issues, it is recommended to streamline specialist consultation protocols, enhance medication delivery systems, expedite lab and radiology turnaround times, and increase staffing levels, particularly nursing personnel, during peak times [20, 21]. Implementing these measures can improve operational efficiency, reduce delays, and ensure timely, high-quality care, ultimately enhancing patient outcomes and satisfaction.

The stages in the ED workflow where bottlenecks are most pronounced are examined in this study, revealing critical areas for process optimization. The Doctor to Decision Time stage is identified as the primary bottleneck, with a staggering 86.1% of times rated as “Unacceptable” and a standardized regression weight of 0.840, representing the most significant delay in the ED workflow. Factors contributing to prolonged LOS in the ED include consulting services, medication administration, laboratory investigations, radiological studies, and non-Saudi nationality [22, 23]. These factors highlight the need for process improvements in these areas. In addition, prolonged physician decision times are influenced by waiting for specialist consultations, holdups in ancillary services, high patient volume and severity, and insufficient nursing personnel [22, 23]. The second major bottleneck is the Triage to Doctor Time stage, with 48.5% of times rated as “Unacceptable” and a standardized regression weight of 0.287, often due to a shortage of beds in the treating areas. Furthermore, the Decision to Disposition Time stage shows that 34.1% of times are rated as “Unacceptable,” with a standardized regression weight of 0.442, indicating significant delays in this part of the process. Lastly, the Registration to Triage stage reveals that 36.0% of cases fall under “Late Registration,” with a standardized regression weight of 0.109, reflecting a substantial issue with timely registration. These findings underscore the importance of prioritizing improvements in the Doctor to Decision Time and Triage to Doctor Time stages to achieve the most significant reductions in overall ED LOS [4, 24].

The analysis reveals significant delays in the patient flow process, particularly affecting CTAS3, CTAS4, and CTAS5 categories. For instance, CTAS3 and CTAS4 patients face substantial delays from Registration to Triage, with mean delays of 53.6% and 29.2%, respectively, compared to CTAS1. Significant delays are also observed in the transition from Triage to Doctor for CTAS4 and CTAS5, with 45.5% of CTAS3 and 66.1% of CTAS4 cases rated as “Unacceptable.” In addition, CTAS5 patients experience the longest delays from doctor to decision, with 90.8% of CTAS3 and 85.2% of CTAS4 cases deemed “Unacceptable.” Finally, the Decision to Disposition phase shows significant delays for CTAS3, CTAS4, and CTAS5, with 34.2% of CTAS3 and 22.4% of CTAS4 cases falling under “Unacceptable.” These findings indicate critical inefficiencies in resource allocation, prioritization practices, and decision-making processes, leading to decreased patient satisfaction and potential adverse health outcomes [23, 25]. To address these issues, it is recommended to increase staffing and resources during peak times, streamline triage protocols, enhance staff training, integrate health information technology for real-time tracking, and establish continuous monitoring systems. Implementing these measures can improve operational efficiency, reduce delays, and ensure timely, high-quality care, ultimately enhancing patient outcomes and satisfaction.

The study identified significant delays in the ED workflow, particularly in the Doctor to Decision Time and Triage to Doctor Time stages, with 86.1% and 48.5% of times rated as “Unacceptable,” respectively, highlighting these as primary bottlenecks. The ANOVA results showed statistically significant differences across triage categories, with CTAS5 experiencing the longest delays, indicating that higher severity cases significantly contribute to prolonged ED stays. Regression analysis further emphasized that Decision to Disposition Time and Doctor to Decision Time have strong positive relationships with total LOS, underscoring their critical impact on overall ED efficiency. These findings imply that consulting services, medication administration, laboratory and radiological investigations, and resource constraints are key factors driving these delays. To address these issues, it is recommended to streamline specialist consultation protocols, enhance medication delivery systems, expedite lab and radiology turnaround times, and increase staffing levels, particularly for nursing personnel, during peak times [26–28]. By implementing these measures, healthcare facilities can reduce delays, improve patient flow, and enhance overall patient outcomes and satisfaction.

Faster bed turnover reduces the Decision to Disposition Time, promptly moving patients who need admission and freeing up ED beds for new arrivals. Adequate staffing alleviates pressure on existing personnel, improves triage and evaluation times, enhances care quality, and supports emergency nursing during peak times. Well-trained nurses handle the dynamic ED environment better, improving patient interactions and reducing delays. Standardized guidelines ensure timely and appropriate treatment for all patients, contributing to a more organized and efficient emergency nursing system. Continuous evaluation and improvement efforts maintain high care standards and operational efficiency, enhancing patient satisfaction. Effective communication and teamwork improve patient flow, reduce bottlenecks, and ensure comprehensive, timely care, which is crucial for effective emergency nursing.

## 5. Conclusions

This study highlights significant inefficiencies in the ED workflow in Saudi Arabia, particularly in the Doctor to Decision Time and Triage to Doctor Time stages, with 86.1% and 48.5% of times rated as “Unacceptable,” respectively. The analysis using ANOVA and regression revealed that higher severity cases (CTAS3, CTAS4, and CTAS5) experience the longest delays, significantly contributing to prolonged ED stays. Factors such as consulting services, medication administration, laboratory and radiological investigations, and resource constraints were identified as major contributors to these delays. To address these critical bottlenecks, it is recommended to streamline specialist consultation protocols, enhance medication delivery systems, expedite lab and radiology turnaround times, and increase staffing levels, particularly nursing personnel, during peak times. Implementing these measures can improve operational efficiency, reduce delays, and ensure timely, high-quality care, ultimately enhancing patient outcomes and satisfaction. Effective emergency nursing management, including faster bed turnover, adequate staffing, standardized guidelines, and continuous evaluation, is crucial in achieving these improvements and maintaining high standards of care in the ED.

## Figures and Tables

**Table 1:** Bottleneck calculation methods in Saudi Arabian emergency department description.

Metric	Starting point	Ending point	Calculation (min)	Performance categories (min)
⁣^∗^Registration to Triage	Patient registration	First triage nurse contact	Ending point–starting point	World class: < 10
				Acceptable: 10–20
				Needs improvement: 20–30
				Unacceptable: > 30

Triage to Doctor Time	First triage nurse contact	First physician contact	Ending point–starting point	World class: < 10
				Acceptable: 10–20
				Needs improvement: 21–40
				Unacceptable: > 40

Doctor to Decision Time	First physician contact	Decision made (admit, discharge, transfer)	Ending point–starting point	World class: < 30
				Acceptable: 30–60
				Needs improvement: 60–90
				Unacceptable: > 90

Decision to Disposition Time	Decision made (admit, discharge, transfer)	Patient leaves ED or moves departments	Ending point–starting point	World class: < 30
				Acceptable: 30–90
				Needs improvement: 90–130
				Unacceptable: > 130

^∗^In some cases, triage and doctor consultations occurred before registration; thus, this period is considered as late registration.

**Table 2:** Performance metrics at each stage of the patient journey.

		Frequency	Percent
Registration to Triage	World class	399	53.0
	Acceptable	47	6.2
	Needs improvement	18	2.4
	Unacceptable	9	1.2
	Late registration	271	36.0
	Total	744	98.8

Triage to Doctor Time	World class	138	18.3
	Acceptable	115	15.3
	Needs improvement	131	17.4
	Unacceptable	365	48.5
	Total	749	99.5

Doctor to Decision Time	World class	32	4.2
	Acceptable	21	2.8
	Needs improvement	52	6.9
	Unacceptable	648	86.1
	Total	753	100.0

Decision to Disposition Time	World class	363	48.2
	Acceptable	90	12.0
	Needs improvement	43	5.7
	Unacceptable	257	34.1
	Total	753	100.0

**Table 3:** Performance metrics by triage categories (CTAS1 to CTAS5).

		Categories					Total (%)
		CTAS1 (%)	CTAS2 (%)	CTAS3 (%)	CTAS4 (%)	CTAS5 (%)	
Registration to Triage	World class	2.0	5.9	25.4	18.1	1.6	53.0
	Acceptable	0.0	0.4	2.9	2.5	0.4	6.2
	Needs improvement	0.1	0.0	0.8	1.3	0.1	2.4
	Unacceptable	0.0	0.3	0.7	0.3	0.0	1.2
	Late registration	3.3	1.3	15.9	9.2	6.2	36.0

Triage to Doctor Time	World class	4.4	5.5	6.3	1.9	0.4	18.3
	Acceptable	0.4	0.9	9.2	3.9	0.9	15.3
	Needs improvement	0.4	0.7	9.7	5.0	1.7	17.4
	Unacceptable	0.5	0.8	21.1	20.8	5.5	48.5

Doctor to Decision Time	World class	0.3	0.7	1.2	1.6	0.5	4.2
	Acceptable	0.4	0.1	0.5	1.1	0.7	2.8
	Needs improvement	0.8	0.7	2.5	2.0	0.9	6.9
	Unacceptable	4.4	6.5	42.0	26.8	6.4	86.1

Decision to Disposition Time	World class	0.7	1.9	20.7	18.9	6.1	48.2
	Acceptable	0.4	0.7	5.8	4.4	0.7	12.0
	Needs improvement	0.1	0.1	3.9	1.2	0.4	5.7
	Unacceptable	4.7	5.3	15.8	7.0	1.3	34.1

**Table 4:** One-way ANOVA comparison of Registration to Triage Times by triage categories.

Comparison	Mean difference	Std. error	Significance (*p* value)	95% confidence interval
CTAS1 vs. CTAS2	−24.00152	6.58768	0.003	−42.0151 to −5.9879
CTAS1 vs. CTAS3	−21.22910	5.31061	< 0.001	−35.7507 to −6.7075
CTAS1 vs. CTAS4	−22.40995	5.44842	< 0.001	−37.3083 to −7.5116

**Table 5:** One-way ANOVA comparison of Triage to Doctor Times by triage categories.

Comparison	Mean difference	Std. error	Significance (*p* value)	95% confidence interval
CTAS1 vs. CTAS4	−56.62140	11.60525	< 0.001	−88.3553 to −24.8875
CTAS2 vs. CTAS3	−49.07644	9.88250	< 0.001	−76.0996 to −22.0533
CTAS2 vs. CTAS4	−76.52595	10.21716	< 0.001	−104.4642 to −48.5877
CTAS2 vs. CTAS5	−50.82500	12.70419	< 0.001	−85.5639 to −16.0861
CTAS3 vs. CTAS4	−27.44951	5.95410	< 0.001	−43.7307 to −11.1684

**Table 6:** One-way ANOVA comparison of Doctor to Decision Times by triage categories.

Comparison	Mean difference	Std. error	Significance (*p* value)	95% confidence interval
CTAS1 vs. CTAS5	76.22017	21.85847	0.005	16.4494 to 135.9909
CTAS3 vs. CTAS4	25.94220	9.40023	0.047	0.2378 to 51.6466
CTAS3 vs. CTAS5	52.32597	15.18075	0.005	10.8151 to 93.8369
CTAS4 vs. CTAS3	−25.94220	9.40023	0.047	−51.6466 to −0.2378
CTAS5 vs. CTAS1	−76.22017	21.85847	0.005	−135.9909 to −16.4494
CTAS5 vs. CTAS3	−52.32597	15.18075	0.005	−93.8369 to −10.8151

**Table 7:** One-way ANOVA comparison of Decision to Disposition Times by triage categories.

Comparison	Mean difference	Std. error	Significance (*p* value)	95% confidence interval
CTAS1 vs. CTAS3	161.25026	32.13215	< 0.001	73.3867 to 249.1138
CTAS1 vs. CTAS4	206.05111	32.96593	< 0.001	115.9077 to 296.1946
CTAS1 vs. CTAS5	211.16477	39.32858	< 0.001	103.6230 to 318.7065
CTAS2 vs. CTAS3	196.95632	28.07227	< 0.001	120.1943 to 273.7183
CTAS2 vs. CTAS4	241.75717	29.02292	< 0.001	162.3956 to 321.1187
CTAS2 vs. CTAS5	246.87083	36.08759	< 0.001	148.1914 to 345.5503

## Data Availability

The data that support the findings of this study are available on request from the corresponding author. The data are not publicly available due to privacy or ethical restrictions.
